# Fluoroscopic parameters in the diagnosis and quantitative assessment of gastric sleeve stenosis

**DOI:** 10.1007/s00464-026-12810-9

**Published:** 2026-05-05

**Authors:** Samuel Tanner, Susie Min, Jessica X. Yu, Sarah Volk, Allison R. Schulman

**Affiliations:** 1 Division of Gastroenterology and Hepatology, University of Michigan, Ann Arbor, MI USA; 2 Department of Internal Medicine, University of Michigan, Ann Arbor, MI USA; 3 Division of Gastroenterology and Hepatology, Oregon Health and Sciences University, Portland, OR USA; 4 Department of Surgery, University of Michigan, Ann Arbor, MI USA

**Keywords:** Fluoroscopy, Gastric sleeve stenosis, Bariatric endoscopy, Impedance planimetry, Sleeve gastrectomy

## Abstract

**Background:**

The diagnosis of gastric sleeve stenosis (GSS) is often delayed due to the lack of readily available objective criteria. The aim of this study was to assess whether fluoroscopic parameters obtained during endoscopy may offer a widely available method to assess GSS.

**Methods:**

This was a retrospective analysis of a single-institution, prospective database of patients who underwent endoscopic evaluation of suspected GSS. Endoscopic severity of GSS, defined by the presence of stenosis and angulation at the level of the incisura, was systemically assessed by a trained bariatric endoscopist. The width of the incisura and angle between the proximal and distal sleeve were measured fluoroscopically. Balloon diameter measurements were obtained at different balloon volumes during impedance planimetry testing and correlated with incisura width.

**Results:**

A total of 63 procedures were included. When stratified by endoscopic impression of stenosis, the mean (± standard deviation) width of the incisura in patients with no stenosis was 17.1 ± 6.5 mm, mild stenosis was 13.1 ± 5.1 mm, moderate stenosis was 12.4 ± 2.6 mm, and severe stenosis was 8.6 ± 2.1 mm (*p* < 0.001). When stratified by endoscopic impression of angulation, the mean (± standard deviation) width of patients with no angulation was 101 ± 29 degrees, mild angulation was 80 ± 28 degrees, moderate angulation was 76 ± 22 degrees, and severe angulation was 68 ± 20 degrees (*p* = 0.21). The width of the incisura correlated with balloon diameter on impedance planimetry (all *p* < 0.05).

**Conclusions:**

Fluoroscopic analysis in GSS supports endoscopic impressions of stenosis severity and complements impedance planimetry measurements. This approach offers a more widely available method for diagnosis of GSS.

Sleeve gastrectomy (SG) has become the preferred bariatric procedure worldwide for the treatment of obesity and its associated comorbid conditions given its high efficacy with limited complication rates [1–4]. During this procedure, a large portion of the stomach is transected along the greater curvature to form gastric sleeve or tube-like structure. This surgery results in reduced stomach size with higher resistance to stretching as well as a decreased production of ghrelin, a hunger-inducing hormone, which together help promote appetite reduction [2]. Recent long-term data has suggested the estimated that patients who undergo SG maintain a total weight loss of 23% at 5 years after surgery [5].

Despite its high efficacy in promoting weight loss, complications such as gastric sleeve stenosis (GSS) can occur. GSS is characterized clinically by obstructive symptoms such as regurgitation, dyspepsia, retrosternal burning, dysphagia, early satiety, abdominal pain, nausea and vomiting and is estimated to occur in 1–4% of patients who undergo SG [6]. In the early post-operative period, it is hypothesized that this is due to localized edema or ischemia that is typically managed conservatively. However, if symptoms persistent further evaluation and treatment are warranted. [7, 8]. The etiology of persistent GSS has been attributed to two possible causes. First, a fixed, or anatomic luminal narrowing can be present typically at the level of the incisura angularis related to fibrosis or scarring from oversewing the staple line or misplacement of the bougie. Second, a twisting or ‘kinking’ of the gastric sleeve can be present likely due to unequal traction of the anterior and posterior gastric walls during stapling resulting in narrowing of the lumen [9, 10].

The diagnosis of GSS can be challenging given the non-specific nature of the clinical presentation as well as the lack of defined objective data. While upper gastrointestinal (GI) series has been proposed as a potential test for GSS, it has been shown to be insensitive for GSS with a low negative predictive value leading to delays in patients receiving treatment [11, 12]. Upper endoscopy can reliably identify luminal narrowing and tortuosity although GSS diagnosis using lumen morphology often requires endoscopist expertise in GSS. We have previously reported the use of automated image analysis and endoluminal functional lumen imaging to quantify GSS severity with promising results. However, these technologies are not readily available in most endoscopy centers [13–16]. Therefore, we sought to develop a widely available technique – routine fluoroscopy during upper endoscopy—that could objectively provide parameters for both the presence and severity of gastric sleeve stenosis.

## Methods

### Patient selection

Consecutive patients presenting for endoscopic treatment for suspected gastric sleeve stenosis from December 2022 to December 2025 were screened for inclusion and exclusion criteria. Inclusion criteria included history of laparoscopic sleeve gastrectomy and symptoms suggestive of sleeve stenosis including reflux, nausea, vomiting, or abdominal pain. Exclusion criteria included protocol violations during the endoscopic procedure and consensus agreement among three authors (S.T., S.M., and A.R.S.) that the fluoroscopic images were inadequate for quantitative interpretation. Reasons for inadequacy of images included overlapping small bowel obscuring the incisura and excessive redundancy of the proximal stomach obscuring the location of the incisura. The protocol for this study was approved by the University of Michigan Institutional Review Board (HUM00210604).

### Endoscopic procedure

All patients were placed in the conventional lazy left-lateral position prior to the endoscopic procedure. A diagnostic upper endoscopic exam was performed by a single, experienced bariatric endoscopist (A.R.S.) to assess gastric sleeve anatomy, including the presence of gastric sleeve stenosis and angulation at the level of the incisura. The endoscopic diagnosis of GSS was based on the perceived ratio of the narrowest to widest part of the luminal diameter as well as gastroscope resistance to passage [15]. Additionally, angulation of stomach at the level of the incisura was assessed. The gastroscope was then withdrawn to the level of the gastroesophageal junction and the gastric sleeve was maximally insufflated using carbon dioxide to obtain fluoroscopic images. All fluoroscopic images were archived in the picture archiving and communication system (PACS). Impedance planimetry measurements including diameter and distensibility at balloon volumes of 30 mL, 40 mL, and 50 mL were then obtained by advancing the impedance planimetry catheter (EndoFlip, Medtronic, Minneapolis, Minnesota, USA) across the incisura of the gastric sleeve in a method previously described [13]. Following the collection of impedance planimetry parameters, patients underwent pneumatic dilation under endoscopic and fluoroscopic guidance if appropriate. All procedures were performed under general anesthesia.

### Data collection

Demographic data including patient age and sex were recorded. Endoscopic reports were reviewed to obtain the qualitative endoscopic impression of stenosis and angulation at the level of the incisura. The grading for stenosis was reported as none (0), very mild or mild (1), mild to moderate or moderate (2), and moderate to severe or severe (3). The grading of gastric sleeve angulation was reported as none (0), very mild or mild (1), mild to moderate or moderate (2), and moderate to severe or severe (3). When impedance planimetry was performed, the maximum diameter (mm) of the narrowest luminal area at each balloon fill volume (30, 40, and 50 mL) was also captured.

### Fluoroscopic parameters

**Fig. 1:**
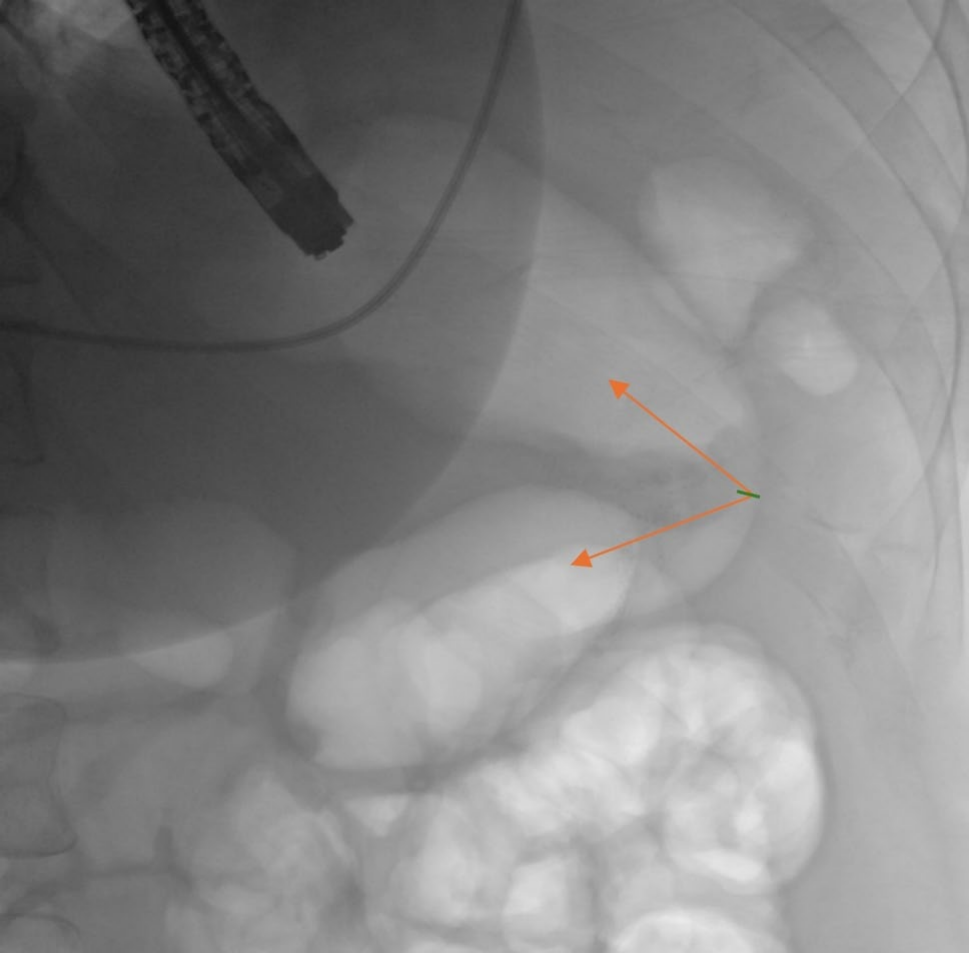
Representative example of fluoroscopic imaging obtained during upper endoscopy in patient with severe stenosis (green line) and angulation (orange arrows) at the level of incisura (Color figure online)

Two authors (S.T. and S.M.) independently analyzed fluoroscopic images and were blinded to procedural information and impedance planimetry values. The width at the incisura and angulation at the level of the incisura were measured using the ruler and angle measurement tools in PACS (Fig. 1). The width of the incisura was defined by identifying the point where the lesser curvature of the stomach showed a change in angle and the ruler was extended perpendicular to this point to the greater curvature. The angulation of the incisura was measured by placing the vertex at the midway point of this line and extending the two arms of the angle towards the gastroesophageal junction and pylorus, respectively. If there was a discrepancy between either the angulation or stenosis by more than 20 degrees or 5 mm, then a third observer (A.R.S.) also reviewed the fluoroscopic images to obtain incisura width and angulation measurements. These cutoffs were chosen as it was felt that this discrepancy likely reflected disagreement on the location of the incisura on fluoroscopic images rather than variations in measurements.

### Statistical analysis

Inter-rater reliability between the two independent measurements for both stenosis and angulation were analyzed using the intraclass correlation coefficient (ICC) using a two-way mixed-effects model. ICC values less than 0.5, between 0.5 and 0.75, between 0.75 and 0.9, and greater than 0.90 are indicative of poor, moderate, good, and excellent reliability, respectively [17]. All data recorded in Microsoft Excel (Microsoft, Redmond, Washington). The mean value of the two (or three) measurements were used for subsequent analysis. Fluoroscopic parameters (either width or angulation, respectively) were stratified by their corresponding endoscopic impression with endoscopic impression (either stenosis or angulation, respectively) being treated as an ordinal variable. The distribution of fluoroscopic parameters between each endoscopic impression were then assessed using a Kruskal–Wallis test. To assess correlation between fluoroscopy and impedance planimetry, the width at the incisura on fluoroscopy was correlated with balloon diameter at each balloon volume using a Spearman correlation test. Statistical analysis was performed in IBM SPSS Statistics v29.0.2.0 (IBM, Armonk, New York).

## Results

### Patient characteristics

**Fig. 2:**
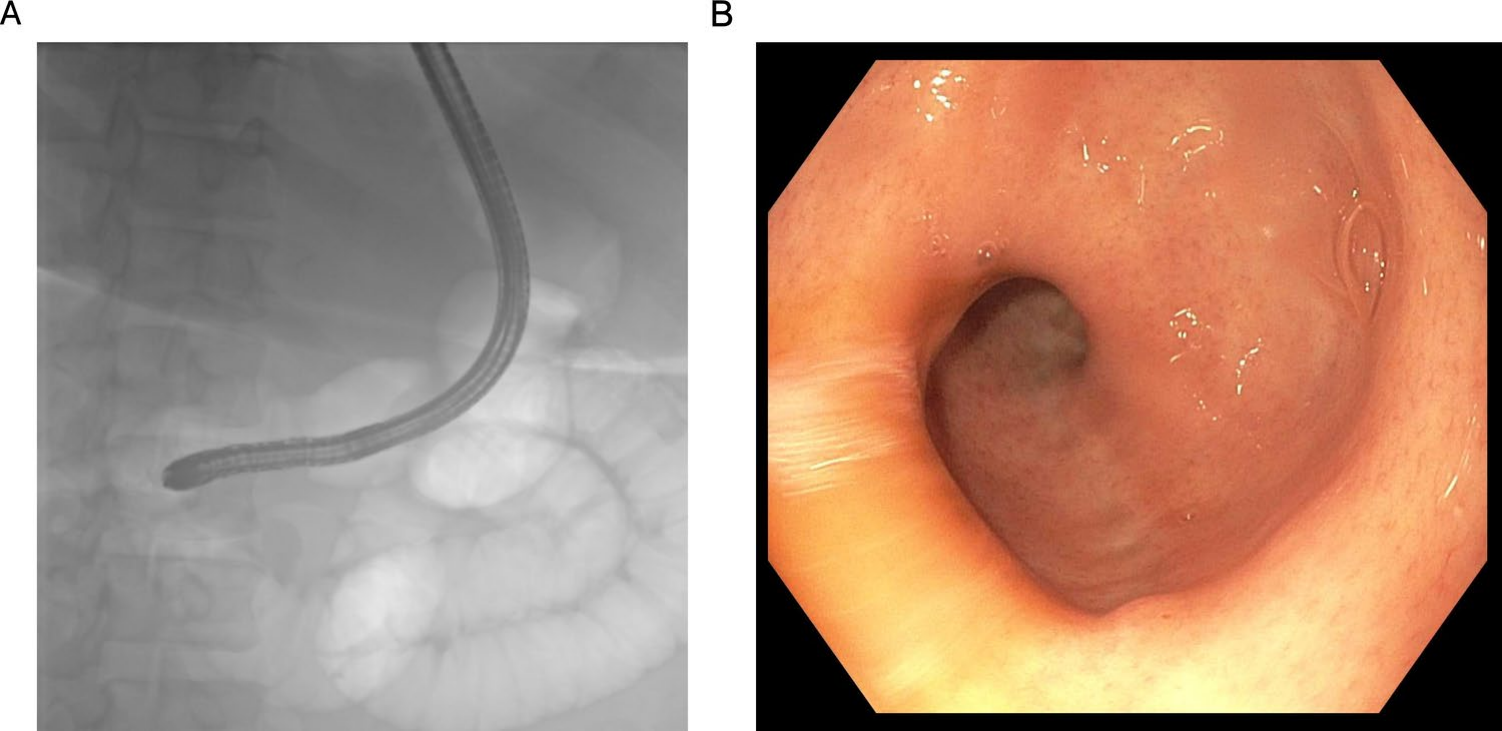
Representative example of both endoscopic (**A**) and fluoroscopic (**B**) views of twisting of the gastric sleeve with fluoroscopic parameters that are unable to be measured reliably

During the study period, a total of 83 upper endoscopies with fluoroscopy with pneumatic dilation were performed for suspected gastric sleeve stenosis. Data from 20 of these procedures were excluded from further analysis either due to variations from procedure protocol described above which would not allow for further analysis (*n* = 6) or inadequate fluoroscopic imaging for obtaining the parameters of interest (*n* = 19) as determined by consensus agreement between three of the authors (S.T., S.M., and A.R.S.) (Fig. 2).

**Fig. 3:**
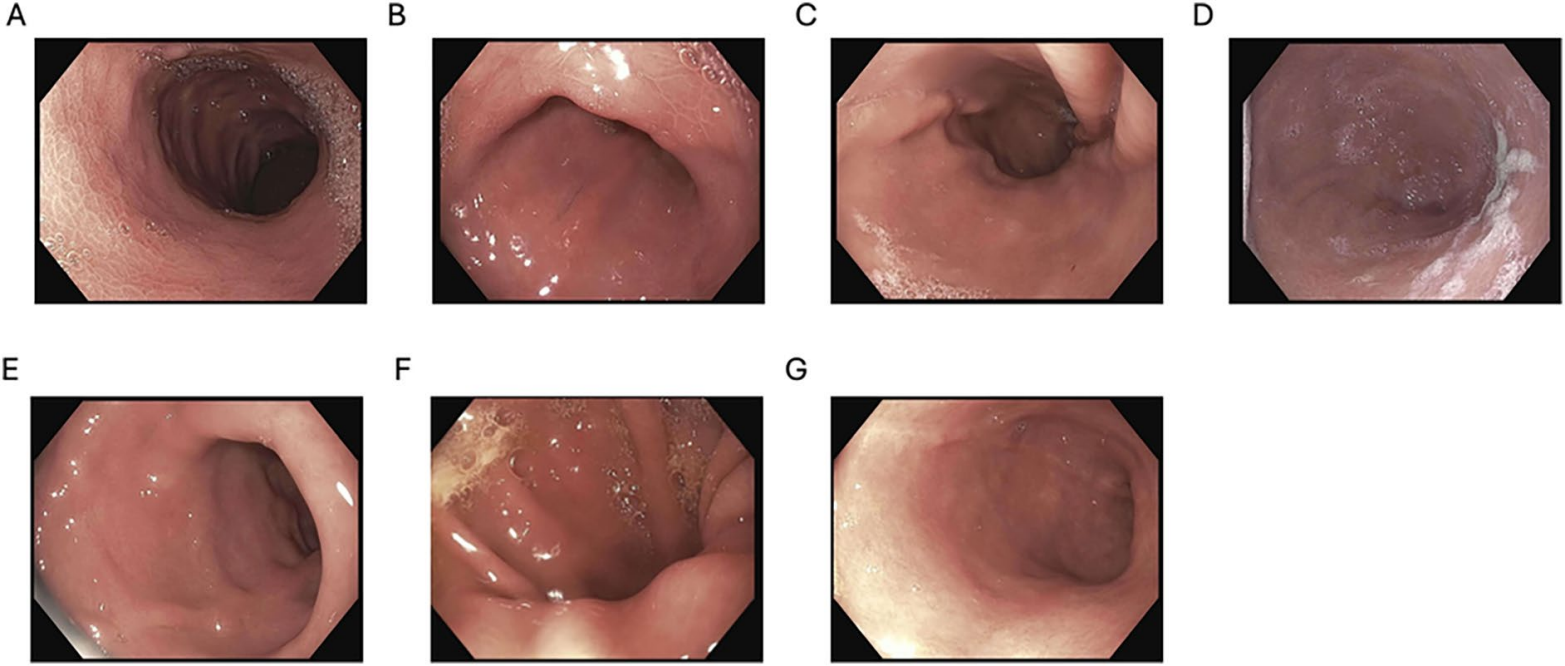
Representative endoscopic images of gastric sleeve stenosis and angulation. **A** No stenosis or angulation. **B** Mild stenosis. **C** Moderate stenosis. **D** Severe stenosis. **E** Mild angulation. **F** Moderate angulation. **G** Severe Angulation

Sixty-three studies were included in our analysis. Mean (± SD) age of the patients included in the cohort was 44.8 (± 10.3) years and 59 (94%) were female. The median time from laparoscopic sleeve gastrectomy to pneumatic sleeve dilation was 51 months (interquartile range: 16 to 100 months). The median total body weight loss percentage was 30% (interquartile range 14 to 39%) from the time of surgery to sleeve dilation. On endoscopic analysis of stenosis, mild, moderate and severe stenosis was seen in 13 (21%), 14 (21%), and 22 (35%) of cases, respectively. 12 cases (19%) had no stenosis and endoscopic impression of stenosis was not reported in 2 cases (3%). On endoscopic analysis of angulation, mild, moderate and severe angulation was seen in 10 (16%), 12 (18%), and 27 (43%) of cases, respectively. 7 (11%) of cases had no angulation and endoscopic impression of angulation was not reported in 7 (11%) of cases. Representative endoscopic images of stenosis and angulation are shown in Fig. 3. Impedance planimetry was performed in 58 procedures (92%).

### Fluoroscopic characteristics

**Fig. 4:**
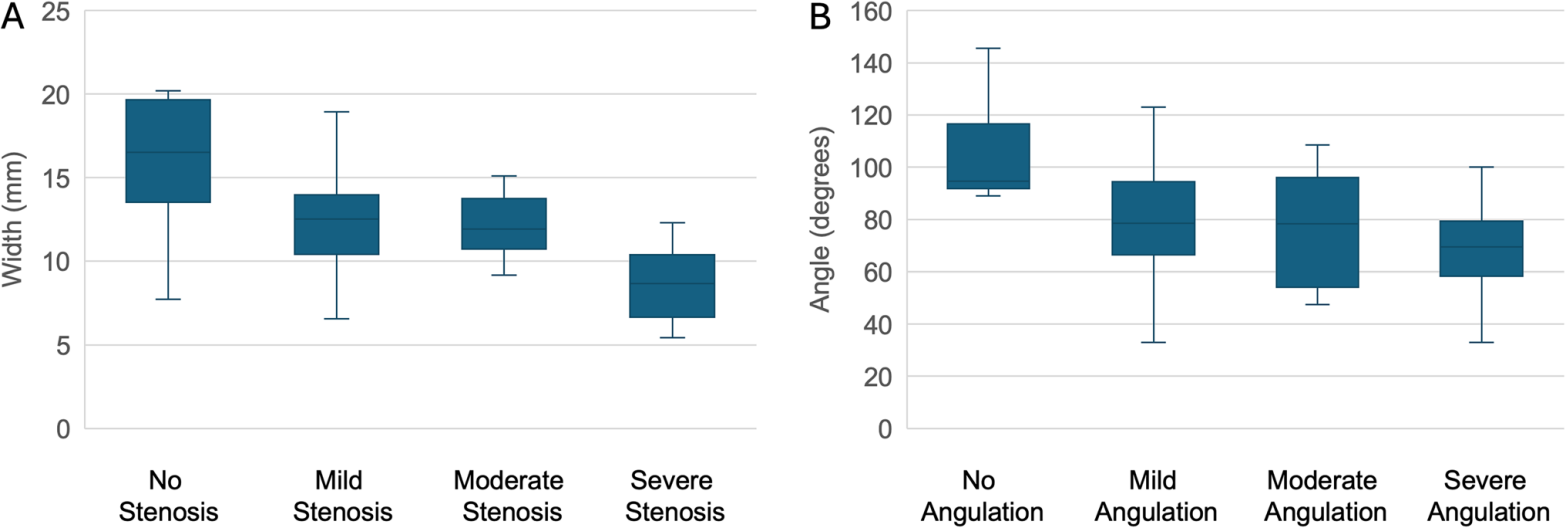
Distribution of fluoroscopic measurements of **A** width and **B** angulation stratified by endoscopic impressions

The mean width at the level of the incisura was 12.2 ± 5.2 mm (range 5.4 to 33.6 mm). The ICC between the two reviewers for the width measurement was 0.83 (95% CI 0.73 – 0.89), suggestive of good reliability. When stratified by endoscopic impression of stenosis, the mean width of patients with no stenosis was 17.1 ± 6.5 mm, mild stenosis was 13.1 ± 5.1 mm, moderate stenosis was 12.4 ± 2.6 mm, and severe stenosis was 8.6 ± 2.1 mm (Fig. 4A *p*< 0.001). The mean angle was 76 ± 25 degrees (range 26 to 146 degrees). The ICC between the two reviewers for the angulation measurement was 0.70 (95% CI 0.20 – 0.91), suggestive of good reliability. When stratified by endoscopic impression of angulation, the mean width of patients with no angulation was 101 ± 29 degrees, mild angulation was 80 ± 28 degrees, moderate stenosis was 76 ± 22 degrees, and severe angulation was 68 ± 20 degrees (Fig. 4B *p* = 0.21).

### Correlation with impedance planimetry

**Fig. 5:**
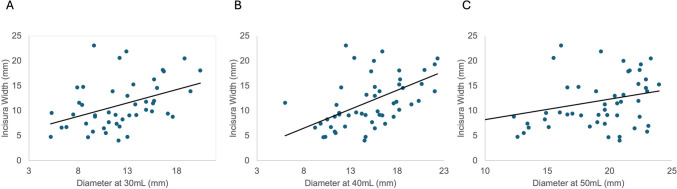
Correlations between fluoroscopic measurements of width and balloon diameter during impedance planimetry testing at **A** 30 mL, **B** 40 mL, and **C** 50 mL balloon volumes

The width of the incisura correlated with the diameter of the balloon at each balloon volume during endoluminal functional probe testing (30 mL: r = 0.41, *p* < 0.01; 40 mL: r = 0.52, *p* < 0.001; 50 mL: r = 0.29, *p* = 0.04, Fig. 5).

## Discussion

This study is the first to report quantitative values for fluoroscopic values for gastric sleeve stenosis obtained during upper endoscopy. We found that the width of the gastric sleeve at the level of the incisura correlates with the endoscopic impression of stenosis severity, providing an objective measure for sleeve stenosis. Additionally, this measurement also correlates with balloon diameter obtained via impedance planimetry further supporting its use in assessing gastric sleeve stenosis.

Stenosis that is observed endoscopically at the level of the incisura likely reflects either an anatomic luminal narrowing related to scarring or fibrosis or a functional luminal narrowing related to sleeve twisting or torsion, or some combination of these processes. We hypothesize that fluoroscopy was a reliable indicator of the degree of stenosis in our study because both these processes likely result in a static narrowing at the level of the incisura which can be observed both endoscopically and fluoroscopically. In comparison, a case series of 17 patients with suspected GSS undergoing evaluation with upper GI series and upper endoscopy found that luminal narrowing secondary to twisting of the gastric sleeve could only be identified endoscopically, but not with an upper GI series [18]. One advantage of fluoroscopic examination during upper endoscopy as compared to an upper GI series is that lazy left lateral positioning may allow for more optimal luminal distension. Additionally, the use of endoscopic insufflation with the endoscope positioned at the gastroesophageal junction promotes maximal distension of the gastric sleeve thereby overcoming the risk of under-distension masking a subtle stricture (and therefore false-negative results) in upper GI series [11]. Using this methodology, we were able to reliably differentiate different degrees of stenosis at the incisura.

While the width at the incisura could reliably provide severity of sleeve stenosis, angulation measurements could not be used as a reliable measure of endoscopic findings of angulation. This is likely due to several factors. First, the endoscopic impression of angulation reflects a complete twisting or torsion of the gastric sleeve rather than a simple kinking at the incisura (which is observed fluoroscopically). This twisting of the gastric sleeve can result in luminal narrowing which has been proposed to be incorporated in endoscopic classification of angulation severity [19]. An additional challenge is that angulation of the gastric sleeve can either be localized twisting typically at the area of the incisura or a more diffuse spiral-shaped staple line [20]. We hypothesize that the angulation-related localized twisting at the incisura can more accurately be captured fluoroscopically than a diffuse spiraling which likely is occurring in several different fluoroscopic planes.

Our study has limitations. First, many procedures did not have adequate imaging to accurately capture fluoroscopic parameters. This is likely due to the fact that there was no standard protocol for fluoroscopic image acquisition. This was a retrospective analysis of real-world practice where the primary role of fluoroscopy was to confirm pneumatic balloon location prior to dilation, and thus these images were not collected for the purposes of this study and therefore not uniformly optimized in terms of patient and x-ray generator position. We hypothesize that changing patient position (including supine position) and rotation of the C-arm could minimize the approximately 1 in 4 inadequate image rate we found in our study. Future prospective studies to standardize this image acquisition approach are currently underway. Furthermore, it is possible that lack of oral contrast used during endoscopic procedures may make it more difficult to differentiate the gastric sleeve from overlaying loops of small bowel or other structures. Additionally, our study lacks fluoroscopic images in patients without obstructive symptoms as this was a requirement for endoscopic and fluoroscopic evaluation. These limitations are balanced by several strengths, including its large sample size, novel application of a widely available technology, and demonstrated high ICC values for both stenosis and angulation measurements, suggesting that images can be reliably interpreted by different observers.

Fluoroscopic analysis in gastric sleeve stenosis objectively confirms endoscopic impressions of stenosis severity and correlates with impedance planimetry measurements. This approach offers a widely available method for the diagnosis of gastric sleeve stenosis that can be used independently or in conjunction with impedance planimetry.
